# Adverse Health Effects of Child Labor: High Exposure to Chromium and Oxidative DNA Damage in Children Manufacturing Surgical Instruments

**DOI:** 10.1289/ehp.1104678

**Published:** 2012-06-01

**Authors:** Muhammad Sughis, Tim S. Nawrot, Vincent Haufroid, Benoit Nemery

**Affiliations:** 1 Lung Toxicology Research Unit, Department of Public Health, KU Leuven, Leuven, Belgium; 2 Centre of Research for Public Health, Lahore, Pakistan; 3 Lahore College of Pharmaceutical Sciences, Lahore, Pakistan; 4 Centre for Environmental Sciences, Hasselt University, Diepenbeek, Belgium; 5 Louvain Centre for Toxicology and Applied Pharmacology (LTAP), Université catholique de Louvain, Brussels, Belgium

**Keywords:** metal exposure, nickel, oxidative DNA damage, Pakistan, Sialkot, surgical instruments

## Abstract

Background: A considerable part of the worldwide production of surgical instruments takes place in Sialkot, Pakistan. Many children work in hazardous conditions in this industry.

Objective: We investigated exposure to metals and possible health effects among children working in surgical instruments manufacturing units compared with schoolchildren from the same city.

Methods: In a cross-sectional study we studied a convenience sample of 104 male children (10–14 years of age) working in surgical instruments manufacturing units and 75 male children of similar age from a school in Sialkot, Pakistan. A respiratory questionnaire was administered, spirometry was performed, and blood pressure was measured. In a spot urine sample, concentrations of metals were measured by inductively coupled plasma mass spectrometry and 8-hydroxydeoxyguanosine (8OHdG, reflecting oxidative DNA damage) by ELISA.

Results: The working children reported more asthma (10% vs. 0%; *p* = 0.005) and dry cough at night (36% vs. 20%; *p* = 0.02) than did the schoolchildren, but there were no significant differences in pulmonary function or blood pressure. The urinary concentration of chromium was 35 times higher in working children [geometric mean, 23.0 µg/L; 25th–75th percentile, 8.38–58.6] than in schoolchildren [0.66 µg/L; 0.38–1.09)], and largely in excess of the occupational Biological Exposure Index for adult workers (25 µg/L). Urinary 8-OHdG concentrations were not significantly higher in working children than in schoolchildren (19.3 vs. 17.6 µg/g creatinine, *p* = 0.4), but were significantly correlated with urinary nickel (*r* = 0.41; *p* < 0.0001) and with a composite index of metal exposure (*r* = 0.46; *p* < 0.0001).

Conclusions: Children working in the surgical instruments manufacturing industry had substantial exposure to several metals, especially chromium and nickel, which are established carcinogens. Exposure to nickel was associated with evidence of increased oxidative DNA damage.

A large fraction of the world’s surgical instruments is manufactured in Sialkot, Pakistan (Bhutta 2006). According to a survey conducted by the Pakistan Federal Bureau of Statistics (1996), 30% of workers in the surgical instruments manufacturing industry were children. Still today, children are employed in labor-intensive and dusty tasks, such as grinding and polishing. Young people are especially vulnerable to many noxious agents (Staessen et al. 2001), and their protection is an important public health challenge (Landrigan and Goldman 2011). Recently, health care providers have developed guidelines intended to ensure that the commodities supplied to them are produced under ethical conditions (Bhutta 2008).

Surgical instruments are made of stainless steel containing varying proportions of chromium (Cr) and nickel (Ni) as well as other metals. Cr is of particular concern because of the toxic, allergenic, and carcinogenic effects of hexavalent Cr (Cr-VI) (Langård and Costa 2007). Ni is also of concern because of its potential for causing allergy and cancer (Klein and Costa 2007).

Measuring concentrations of occupational agents in blood or urine of workers is a well-established technique to assess personal exposure at work, and such biomonitoring is useful when workplaces cannot be accessed easily to take environmental samples. Urinary concentrations of metals reflect ongoing or cumulative exposure, depending on their toxicokinetics (Lauwerys and Hoet 1993). The use of urine biomarkers is ideal for field studies in children because urine collection is noninvasive and because urinary biomonitoring provides a convenient method to assess recent exposure to many metals, including Cr. We acknowledge that the extent to which urinary concentrations of metals correctly reflect past or current individual exposure depends on the kinetic behavior of each specific metal.

Exposure to redox-active transition metals leads to increased production of reactive oxygen species, which in turn may cause oxidative DNA lesions that may contribute to the pathology of various diseases (Marnett 2000). 8-Hydroxydeoxyguanosine (8-OHdG) is one of the base modifications resulting from oxidative DNA lesions (Tsuzuki et al. 2007) and its abundance is therefore a reflection of oxidant-induced alterations in the genetic material. The repair process of DNA lesions leads to the excretion of 8-OHdG in the urine (Marnett 2000). Hence, the urinary concentration of 8-OHdG is considered to be a suitable biomarker of oxidative stress (Valavanidis et al. 2009).

The objectives of our study were to quantify metal exposure through urinary biomonitoring in children working in the surgical instrument manufacturing industry and to assess possible health effects. Respiratory effects were anticipated because of the presumed higher exposure to dust containing irritant (and possibly asthmogenic) metals. Blood pressure (BP) was chosen as an easily measurable and health relevant cardiovascular end point. Having found evidence of very high exposure to metals (especially Cr), we then tried to verify—using the only available material (i.e., urine)—whether this high exposure to metals with established carcinogenic and pro-oxidant properties would be reflected in oxidative DNA damage, which we assessed by measuring urinary 8-OHdG, as done by others (Wong et al. 2005).

## Methods

*Study groups.* The study was conducted in three campaigns: Working children were studied in April 2009 (*n* = 34), in February–April 2010 (*n* = 59), and in August 2010 (*n* = 11), and all the schoolchildren (*n* = 75) were studied in August–September 2010. The study was conducted in accordance with the Declaration of Helsinki Ethical Principles for Medical Research Involving Human Subjects (World Medical Association 2008) and was approved by Lahore College of Pharmaceutical Sciences. For schoolchildren, parents provided verbal informed consent, which was affirmed in writing by the children themselves. Working children were recruited via their employers, who were first approached through private contacts. Working children who could write their names provided a signed informed consent form, and for those who could not do so, interviewers wrote the names of children on the consent form. Working children were examined during working hours. Participants were free to withdraw at any time during the study, no dangerous or invasive measurements were performed, and confidentiality of the personal results was guaranteed.

The sole inclusion criteria were age (10–14 years) and willingness to participate. Of 117 working children approached, 104 (89%) agreed to participate. Of the 79 schoolchildren who were asked to participate, 75 (95%) gave written informed consent.

*Questionnaire and anthropometric measures.* Tobacco smoking (active and passive), intake of medicines, exposure to biomass smoke, and social class of parents were assessed by a pretested questionnaire adapted from the questionnaire of the International Study of Asthma and Allergies in Childhood (ISAAC 2004) administered in Urdu (the national language of Pakistan) by trained staff in face-to-face interviews. Social class was defined by paternal education as low (≤ 8 years of education), middle (8–12 years), and high (12 to ≥ 16 years). Age was calculated as years since date of birth (schoolchildren) or approximate years as given by contractor. Standing height and weight were measured without shoes; body mass index (BMI) was calculated by dividing weight by the square of height.

*Exposure assessment.* A spot urine sample was collected, after washing hands, in a sterile polystyrene container (100 mL). Samples were stored in a domestic freezer and later shipped by courier service to Leuven, Belgium. Creatinine level was determined using a Beckman Synchron LX 20 analyzer (Beckman Coulter GmbH, Krefeld, Germany); urine samples with creatinine values < 0.3 g/L were excluded (Cocker et al. 2011). The concentrations of 20 metals were measured by inductively coupled argon plasma mass spectrometry (ICP-MS) with an Agilent 7500ce instrument as described elsewhere (Banza et al. 2009). The metal composition (except for iron) of one raw surgical instrument (scissor), taken from one of the studied workshops, was assessed similarly by ICP-MS following acid solubilization of four different small pieces obtained from the instrument.

*8-OHdG analysis.* Urinary 8-OHdG was measured in urine supernatants after centrifugation (2,000 × *g* for 10 min) using an enzyme-linked immunosorbent assay (ELISA; Japan Institute for the Control of Aging). The coefficient of variation between duplicate samples (*n* = 127) was 7%. Urinary 8-OHdG was not measured in the first 34 children because their urine had not been shipped on dry ice.

*Pulmonary function.* Lung function was measured, with the subject seated and without a noseclip, using a pocket spirometer (Spirobank MIR, Rome, Italy). The Spirobank was linked with a portable computer allowing visualization of the maneuvers for the measurements in schoolchildren, but not in the working children, where the maneuvers were stored and downloaded later. The spirometry data obtained from the first 34 working children were discarded because their quality was insufficient and, for logistic reasons, spirometry was not measured among the 11 working children studied in the third campaign. The best values based on forced expiratory time (FET) and shape of the slope from 3–6 maneuvers was retained for analysis. Children rarely achieve the recommended (Miller et al. 2005) FET of 6 sec (Arets et al. 2001), and we therefore accepted maneuvers with a FET of at least 1 sec. Spirograms and flow-volume loops were checked for quality by an experienced technician from the University Hospital Leuven. Predicted values were derived from Zapletal et al. (1977) and Boskabady et al. (2004).

*BP.* Five consecutive readings of BP were taken (after 5 min sitting rest) using an automated BP instrument (Stabil-O-Graph; IEM, Stolberg, Germany) with a cuff suitable for < 33-cm arm circumference, according to the guidelines of the European Society of Hypertension (O’Brien et al. 2003). The mean of the five measurements was used for analysis and to classify participants as having normal blood pressure (systolic < 120 mmHg and diastolic < 80 mmHg) or prehypertension (systolic 120–139 mmHg or diastolic 80–89 mmHg) (Chobanian et al. 2003).

*Statistical analysis.* For database management and statistical analysis, we used SAS software (version 9.1; SAS Institute Inc., Cary, NC, USA). Only metals for which at least half the samples had concentrations above the limits of detection (LOD) were retained for analysis. For concentrations below the LOD, half the LOD was assigned for the calculation of means. Data that were not normally distributed were log transformed. We first compared unadjusted means and proportions between schoolchildren and working children with analysis of variance and Fisher’s exact test, respectively. We calculated dose–effect relationships between urinary metals and blood pressure, lung function indices, 8-OHdG, and physician-diagnosed asthma, while adjusting for potential *a priori* selected confounders and/or covariates, by use of multiple-linear regression, or multiple-logistic regression (asthma). Effect sizes and odds ratios with 95% confidence intervals (CIs) were estimated for a doubling of urinary metal concentrations. We also derived a composite metal exposure index, based on the first component of a principal-components analysis of the urinary concentrations of those 11 metals [aluminum (Al), vanadium (V), Cr, manganese (Mn), Ni, copper (Cu), molybdenum (Mo), cadmium (Cd), tin (Sn), antimony (Sb), and uranium (U)] whose levels were significantly higher in the working children than in schoolchildren. The level of significance was set at *p* < 0.05 (two-sided).

## Results

*General characteristics.* Although the schoolchildren were recruited within the same age range as the working children, the two groups turned out not to be perfectly matched with respect to age, so they varied in height and weight (but not BMI) (Table 1). The working children came from 21 different workshops (1–5 children examined per workshop). Socioeconomic status (defined by paternal education) was lower among the working children than among the schoolchildren. More than half (60%) of the working children had never been to school, and 36% were studying part time. All participants except two working children claimed to be nonsmokers. However, 68% of the working children and 45% of the schoolchildren had a smoker in the family. Among working children, 45% reported the use of wood for cooking at home, compared with none of the schoolchildren (*p* < 0.0001).

**Table 1:** Characteristics of schoolchildren and children working in surgical instruments manufacturing units [mean ± SD or *n* (%)].

Characteristic	Schoolchildren (*n* = 75)	Working children (*n* = 104)	*p*-Value
Age (years)	11.5 ± 1.4	12.4 ± 1.3	< 0.0001
Height (cm)	134.1 ± 10.1	141.0 ± 8.6	< 0.0001
Weight (kg)	29.6 ± 8.3	35.0 ± 6.6	< 0.0001
BMI (kg/cm2)	16.3 ± 3.5	16.9 ± 3.0	0.2
Paternal education < 8 years	44 (58)	97 (92)	< 0.0001

*Working conditions.* The information regarding working conditions, as derived from observations and from interviews, can be summarized as follows: Most children worked 6 days/week; 31% reported working for 8, 29% for 10, and 34% for 12 hr/day. None of the children used any personal protective equipment to reduce potential exposures. Almost all children complained of joint or muscle pains due to bad posture at work. Some children from nearby villages were housed on the working premises, so they were also exposed to dust while not working. In general, the workshops were confined, with poor ventilation and lighting. In winter, the indoor environment appeared relatively better with regard to suspended dust and temperature, but in summer ceiling fans caused the dust to be continuously resuspended.

*Urinary metal concentrations.* Table 2 presents urinary metal concentrations as micrograms per liter and Table 3 presents them as micrograms per gram creatinine after excluding samples with values < 0.3 g/L (*n* = 8). Urinary creatinine concentration was significantly lower among working children than among schoolchildren (geometric means of 0.83 vs. 1.08 g/L, *p* = 0.0008) (Table 3). All metals were significantly correlated with each other, except for Cr vs. zinc (Zn) (*p* = 0.2) and Cr vs. barium (Ba) (*p* = 0.7) [see Supplemental Material, Table S1 (http://dx.doi.org/10.1289/ehp.1104678)]. Geometric mean urinary concentrations of five elements (V, Cr, Ni, Sn, Sb) were significantly higher in working children both when expressed as micrograms per liter and as micrograms per gram creatinine; for seven additional elements [Al, Mn, Cu, selenium (Se), Mo, Cd, U], values were higher in working children only when expressed per gram creatinine. Urinary concentrations of four elements [Zn, arsenic (As), Ba, lead (Pb)] were significantly lower in working children than schoolchildren before, but not after, correction for creatinine. Eleven of the 12 metals with higher urinary concentrations in working children than in schoolchildren were subjected to a principal-component analysis to derive a composite exposure variable for the working children. (Se was excluded because Se was unlikely to be linked with occupational exposure and because adverse effects are more likely to occur with low than high Se levels.) The loading values of the different metals to determine this variable are given in Supplemental Material, Table S2 (http://dx.doi.org/10.1289/ehp.1104678); the group variable (working vs. schoolchildren) explained 27% of the variance in this composite exposure index.

**Table 2:** Metal concentrations (µg/L) in urine [geometric means (25th–75th percentiles)] from schoolchildren and children working in surgical instruments manufacturing units.

Urinary metals	Schoolchildren (n = 75)	Working children (n = 102)b	p-Valuea	Reference values according to NHANES (age group)c				
				6–11 years	12–19 years	6–88 years
Al	24.7 (16.20–33.0)	23.4 (11.60–37.6)	0.8
V	0.86 (0.71–1.08)	1.09 (0.82–1.38)	0.002
Cr	0.66 (0.38–1.09)	23.0 (8.38–58.6)	< 0.0001	0.13 (0.28–0.70)
Mn	2.96 (2.04–3.92)	3.33 (1.78–5.41)	0.3	0.53 (1.84–3.33)
Co	1.08 (0.67–1.97)	0.98 (0.61–1.84)	0.4	0.45 (0.75–1.68)	0.46 (0.74–1.60)
Ni	6.01 (4.25–8.48)	7.45 (5.48–10.6)	0.01
Cu	17.5 (13.2–22.3)	16.3 (11.50–25.1)	0.5
Zn	393 (264–599)	278 (143–518)	0.0008
As	19.9 (14.9–27.4)	14.1 (9.0–23.0)	0.001	7.08 (10.9–46.9)	8.55 (15.2–46.1)
Se	44.5 (34.0–59.6)	39.8 (26.4–60.5)	0.2
Mo	129.0 (89.5–182.0)	167.0 (97.4–308)	0.06	62.20 (108.0–181.0)	52.50 (87.3–143.0)
Cd	0.56 (0.41–0.76)	0.48 (0.31–0.78)	0.1	0.07 (0.12–0.31)	0.12 (0.20–0.40)
Sn	0.05 (0.01–0.20) [17.5%]	0.16 (0.07–0.40) [5.4%]	< 0.0001	3.13 (6.02–20.0)
Sb	0.04 (0.009–0.11) [23.4%]	0.16 (0.11–0.29) [4.0%]	< 0.0001	0.09 (0.16–0.31)	0.10 (0.15–0.29)
Ba	5.10 (3.05–7.73)	3.27 (1.94–5.74)	0.0004	2.21 (4.76–11.80)	2.16 (4.11–9.63)
Pb	5.70 (3.98–8.33)	4.27 (2.80–6.31)	0.01	0.80 (1.35–3.33)	0.60 (0.92–1.86)
U	0.086 (0.057–0.125)	0.089 (0.045–0.146)	0.5	0.008 (0.012–0.028)	0.010 (0.015–0.038)
Values in square brackets [ ] indicate percentages of samples below the LOD (not indicated if all values are above the LOD). ap-Values from a model adjusted for age, height, and weight. bUrine sample could not be obtained from two working children. cU.S. National Health and Nutrition Examination Survey: geometric mean (75th–95th percentile). Values for 6–11 years and 12–19 years from Centers for Disease Control and Prevention (2009); values for 6–88 years from Paschal et al. (1998).												

**Table 3:** Metal concentrations (µg/g creatinine) in urine [geometric means (25th–75th percentiles)] from schoolchildren and children working in surgical instruments manufacturing units.

Schoolchildren (*n* = 74)	Working children (*n* = 94)	Reference values according to NHANES (age group)^b^				
		Urinary metals	*p*-Value^a^	6–11 years	12–19 years	6–88 years
Creatinine (g/L)	1.08 (0.80–1.30)	0.83 (0.5–1.03)	0.0008
Al	24.8 (17.4–28.0)	31.9 (19.5–47.4)	0.01
V	0.85 (0.65–1.17)	1.43 (0.88–2.14)	< 0.0001
Cr	0.66 (0.40–0.82)	30.1 (12.3–78.2)	< 0.0001	0.12 (0.23–0.60)
Mn	2.94 (1.90–3.97)	4.22 (2.04–7.12)	0.02	0.48 (1.16–2.42)
Co	1.09 (0.70–1.76)	1.30 (0.74–2.10)	0.1	0.53 (0.69–1.30)	0.33 (0.50–0.95)
Ni	6.02 (4.54–7.90)	9.87 (6.76–13.8)	< 0.0001
Cu	17.6 (14.9–20.2)	22.1 (16.0–31.0)	0.004
Zn	394.0 (274.0–554.0)	402.0 (294–561)	0.7
As	20.0 (17.3–24.0)	19.2 (14.2–26.0)	0.8	8.25 (11.7–40.1)	6.11 (9.66–27.8)
Se	44.7 (36.8–56.0)	54.9 (42.8–73.2)	0.006
Mo	131.0 (96.2–190)	234.0 (145–409)	< 0.0001	72.5 (101–160)	37.5 (53.2–81.0)
Cd	0.56 (0.43–0.72)	0.66 (0.49–1.0)	0.05	0.09 (0.12–0.31)	0.08 (0.12–0.23)
Sn	0.05 (0.01–0.14)	0.20 (0.11–0.44)	< 0.0001	2.84 (5.01–16.1)
Sb	0.04 (0.01–0.10)	0.22 (0.16–0.37)	< 0.0001	0.11 (0.16–0.33)	0.07 (0.10–0.19)
Ba	5.13 (3.55–7.57)	4.44 (2.58–6.0)	0.3	2.58 (4.45–10.3)	1.54 (2.60–6.47)
Pb	5.70 (4.10–8.0)	5.68 (3.85–8.42)	0.6	0.92 (1.45–3.47)	0.43 (0.62–1.23)
U	0.085 (0.061–0.101)	0.124 (0.064–0.187)	0.009	0.009 (0.013–0.033)	0.007 (0.01–0.034)
Values in µg/g creatinine after exclusion of creatinine values < 0.3 g/L ap-Values from a model adjusted for age, height, and weight. bU.S. National Health and Nutrition Examination Survey: geometric mean (75th–95th percentile). Values for 6–11 years and 12–19 years from Centers for Disease Control and Prevention (2009); values for 6–88 years from Paschal et al. (1998).												

The metal composition of the one surgical instrument analyzed confirmed the presence of high concentrations of Cr (12.5%) with lower concentrations of other metals, including Ni (0.16%).

*Respiratory symptoms and spirometry.* Regarding respiratory symptoms, 36% of working children responded positively to the question “Within the last year, have you had a dry cough at night, apart from a cough associated with a cold or chest infection?” compared with 20% of the control group (*p* = 0.02). Ten working children (10%) reported having physician-diagnosed asthma, compared with none of the schoolchildren (*p* = 0.005). Thirteen working children (33%) reported wheezing compared with 24 (36%) of the control group (*p* = 0.7).

After exclusion of unacceptable tests, we had spirometry data from 39 working children and 67 schoolchildren (Table 3). Forced vital capacity (FVC) and forced expiratory volume in 1 sec (FEV_1_) were substantially higher in the working children than in schoolchildren when we used the predicted values of either Zapletal et al. (1977) or Boskabady et al. (2004). However, the maximal expiratory flows did not differ between the two groups. Of the 8 schoolchildren who had unacceptable spirometry, only 1 reported respiratory symptoms (cough); of the 65 working children without spirometry or with unacceptable spirometry, 21 reported respiratory symptoms, including wheezing in 13. FVC and FEV_1_ were weakly (but positively) associated with urinary Cr (0.07 L; 95% CI: 0.04, 0.10 for FVC; 0.06 L; 95% CI: 0.04, 0.09 for FEV_1_ for a doubling of creatinine corrected Cr) and nonsignificantly with the composite exposure variable (0.02 L; 95% CI: –0.01, 0.06 for FVC; 0.01 L; 95% CI: –0.01, 0.04 for FEV_1_, for a 1-unit change in the composite index) after adjustment for height [see Supplemental Material, Table S3 (http://dx.doi.org/10.1289/ehp.1104678)].

*Blood pressure.* Mean systolic BP was higher (*p* = 0.06) by 2.9 mmHg in working children compared with schoolchildren, but the difference decreased after adjustment for age (Table 4). Mean diastolic BP was lower in working children compared with schoolchildren (1.0 mmHg and 1.7 mmHg lower before and after adjustment for age, respectively), but the difference was not statistically significant (Table 4). None of the children had hypertension; 11 (16%) schoolchildren and 19 (18%) working children had prehypertension (systolic 120–139 mmHg or diastolic 80–89 mmHg) (*p* = 0.7) (Chobanian et al. 2003). Urinary metals, including Pb, were not significantly associated with either systolic or diastolic blood pressure [see Supplemental Material, Table S3 (http://dx.doi.org/10.1289/ehp.1104678)].

**Table 4:** Clinical parameters of schoolchildren and children working in surgical instruments manufacturing units.

Characteristics	Schoolchildren	Working children	*p*-Value
Blood pressure (mean ± SD)
n	68a	104
Systolic BP (mmHg)	110.0 ± 10.4	112.8 ± 7.7	0.06
Diastolic BP (mmHg)	68.2 ± 10.1	67.0 ± 7.5	0.4
Blood pressure [age-adjusted mean (95% CI)]
Systolic BP (mmHg)	111.1 (109.0, 113.2)	112.0 (110.4, 113.7)	0.4
Diastolic BP (mmHg)	68.5 (66.3, 70.6)	66.8 (65.1, 68.5)	0.2
Pulmonary function (mean ± SD)b
n	67	39
Zapletal et al. 1977
FVC (% predicted)	97.2 ± 17.0	112.8 ± 28.0	0.002
FEV1 (% predicted)	101.9 ± 16.0	117.9 ± 28.0	0.001
FEV1/FVC × 100	88.5 ± 6.2	87.8 ± 5.6	0.5
Boskabady et al. 2004
FVC (% predicted)	98.1 ± 15.0	109.9 ± 22.0	0.005
FEV1 (% predicted)	98.1 ± 14.0	108.5 ± 21.0	0.008
PEF (% predicted)	116.1 ± 18.0	115.2 ± 22.0	0.8
FEF25 (% predicted)	105.2 ± 21.0	104.7 ± 17.0	0.9
FEF50 (% predicted)	87.4 ± 22.0	89.8 ± 20.0	0.5
FEF75 (% predicted)	73.1 ± 24.0	82.7 ± 28.0	0.06
Abbreviations: FEF25, 50, 75, forced expiratory flow 25%, 50%, 75% of FVC; PEF, peak expiratory flow. aBlood pressure could not be measured in seven schoolchildren. bPercent predicted according to regression equations published by Zapletal et al. (1977) or Boskabady et al. (2004), except in the case of FEV1/FVC where actual values are shown.						

**Figure 1:**
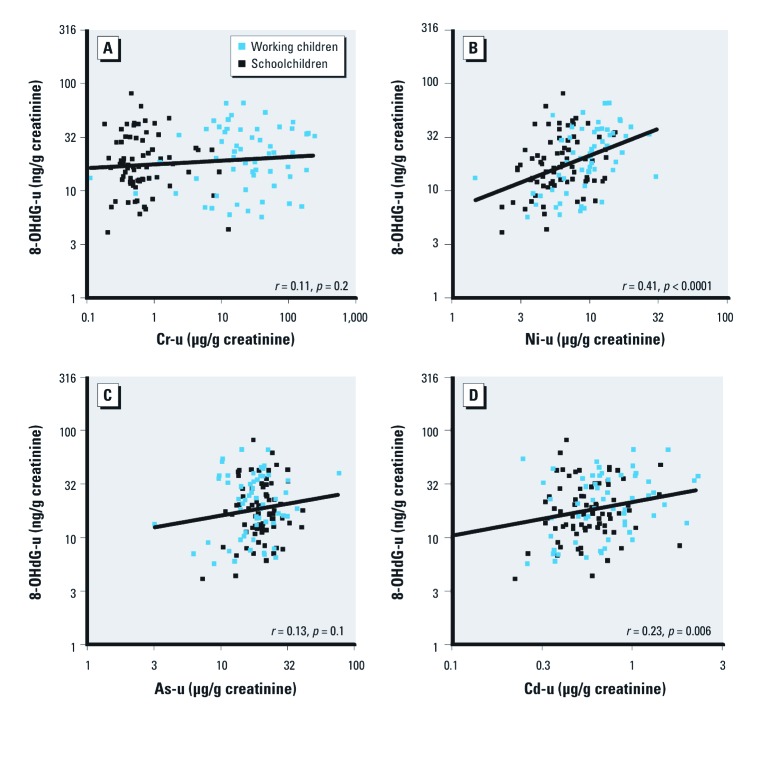
Association of 8-OHdG in urine with urinary (-u) concentrations of Cr (*A*), Ni (*B*), As (*C*), and Cd (*D*). Both axes are logarithmically scaled. The data points are the individual actual values.

**Figure 2:**
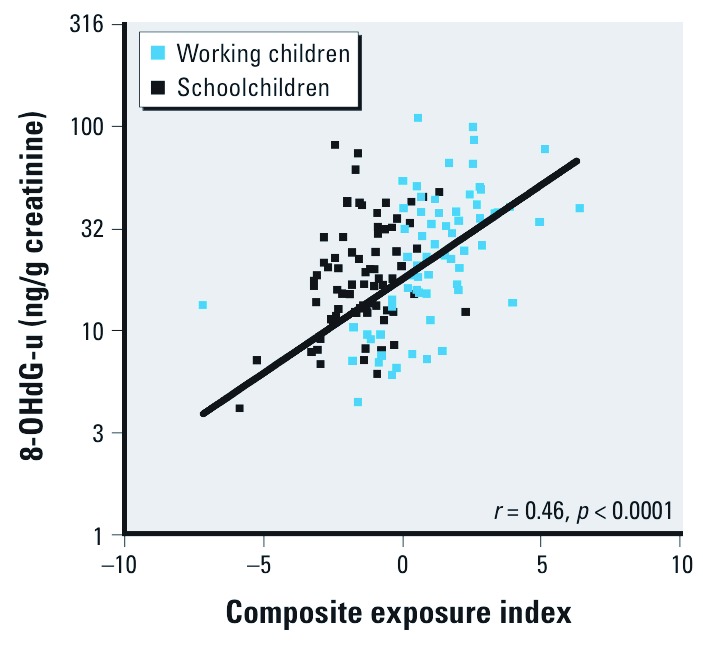
Association of 8-OHdG in urine (-u) with a composite metal exposure index. The *y*-axis is logarithmically scaled. The data points are individual values.

*Oxidative DNA damage.* Urinary concentrations of 8-OHdG did not differ significantly (*p* = 0.4) between the working children (geometric mean 19.3; 25th–75th percentile: 11.0–35.0 µg/g creatinine) and the schoolchildren (17.6; 25th–75th percentile: 12.1–28.0 µg/g creatinine). However, urinary 8-OHdG was positively associated with urinary Ni and Cd before (Figure 1) and after adjustment for age, height, and weight (1.40 µg/g creatinine; 95% CI: 1.23, 1.61, and 1.22 µg/g creatinine; 95% CI: 1.05, 1.43 for a doubling of Ni and Cd, respectively) [see Supplemental Material, Table S3 (http://dx.doi.org/10.1289/ehp.1104678)]. Correlation coefficients for 8-OHdG and urinary metal concentrations ranged from 0.07 to 0.41 (data not shown). The urinary 8-OHdG concentration was highly significantly correlated with the composite metal exposure index (Figure 2).

## Discussion

The International Labor Organization (ILO) reported that, in 2008, an estimated 52.8 million children 5–14 years of age were in hazardous work, in various sectors, especially in the developing world (ILO 2011). The ILO report gives examples of surveys of children engaged in hazardous work and highlights the negative psychosocial impact of child labor, as well as the higher vulnerability of children and adolescents to accidents and toxic exposures. However, only a few scientific publications have reported actual exposures to toxic agents and their adverse health effects in working children. The novelty of our study is that we have documented a substantial exposure to metals in children working in the surgical instruments manufacturing industry in Pakistan and that we have attempted to assess possible health consequences of such work.

The surgical instruments industry of Sialkot has received attention recently, because it is the main primary site of the production of surgical instruments in the world, even for instruments claimed to be “made in Germany” (Bhutta 2006). Although there have been reports about this subject (Centre for the Improvement of Working Conditions and Environment 2003; ILO 2004), to our knowledge, no formal scientific occupational health studies have been published from this sector. The strengths of our study are that we included a large number of working children, that we compared them with schoolchildren from the same area, that we measured individual exposure to metals using biomonitoring, and that we included a biomarker of oxidative DNA damage and clinical data. The main limitations are that it was a cross-sectional study, that recruitment may have been biased, and that respiratory end points were difficult to assess in many of the working children.

A well-known drawback of cross-sectional surveys of working populations is the “healthy worker effect” that occurs because healthy subjects are more likely to be recruited and to remain at work (Pearce et al. 2007). A lesser-known selection bias is the “healthy workshop effect” that occurs because workplaces with the poorest working conditions are often the least accessible for occupational health studies (Nemery et al. 1992). We do not know the extent to which these selection biases influenced our study; but if they were present, they would have tended to diminish any differences between the working children and the schoolchildren. The working children were recruited without any sampling strategy other than accessibility, so we do not know how representative they were for the entire children population in this industry. Nevertheless, our findings indicate high metal exposures in the sector. For obvious reasons, the schoolchildren came from families with a higher socioeconomic status, but we do not believe that this invalidates our conclusions regarding the exposure of the working children.

The healthy worker effect possibly explains the counterintuitive observation that mean FEV_1_ and FVC were significantly and substantially better in the working children, with even a positive (though nonsignificant) association with the composite exposure index. However, a possible explanation is that the age of (some) working children was in fact higher than reported by their employers (although one would expect the opposite tendency). However, the differences were similar if the pulmonary function values were corrected only for height (and not age). Another possible explanation is that we were able to obtain acceptable spirometry in only 39 working children (37%), whereas we had good spirometry in > 90% of the schoolchildren. We attribute the low success of spirometry among the working children to their unfamiliarity with medical examinations, but also to the difficulty of performing spirometry in the field without visual control of the maneuvers. Other researchers have experienced similar difficulties (Zanconato 2005). In adults, poor spirometric performance may reflect poor respiratory health (Ng’Ang’a et al. 1992). The prevalence of self-reported respiratory symptoms was higher among working children than among schoolchildren. Although the responses to the questionnaire might be less reliable among the working children, it is reasonable to attribute this excess in respiratory symptoms and asthma among the working children to their high dust exposure. Longitudinal studies with more detailed clinical data are needed to determine whether some children will develop asthma as a consequence of their occupational exposures. The higher prevalence of respiratory symptoms among the working children does not necessarily contradict the spirometry findings, because several children who reported respiratory symptoms, including wheezing or asthma, did not have satisfactory pulmonary function measurements, possibly because of their respiratory problems. In addition, respiratory symptoms and asthma may be present without measurable alterations in spirometry. Furthermore, it is conceivable that pulmonary function was not yet affected after only a few years’ work.

We found no significant differences in systolic and diastolic pressure between the two groups. Of all the metals that we measured, Pb is the one that has been most closely linked with hypertension (Nawrot et al. 2002), but the working children and schoolchildren did not differ with respect to creatinine-corrected Pb concentrations in urine, although chronic Pb exposure is better assessed by measuring Pb in blood (Gulson et al. 1998).

Our study’s strongest finding concerns the high exposure of the working children to metals. Even among the schoolchildren, (geometric) mean urinary concentrations of most elements were above the 75th percentile or even the 95th percentile (e.g., Cd, Pb) of mean values based on the U.S. National Health and Nutrition Examination Survey (NHANES) (Tables 2, 3) (Centers for Disease Control and Prevention 2009; Paschal et al. 1998). Presumably, these relatively high urinary metal concentrations reflect a more metal-polluted environment in Sialkot than in the United States (Malik et al. 2010), and this reinforces the need to use local reference data to make meaningful comparisons. The working children had significantly higher concentrations of several metals than the schoolchildren, and the increases were observed for metals that are typically present in stainless steel (as confirmed in our, admittedly limited, assessment of an instrument taken from one of the studied workshops). Thus, Cr exhibited the highest contrast with urinary concentrations that were 35 to 45 times higher (for values in micrograms per liter and micrograms per gram creatinine, respectively) among working children compared with the schoolchildren. The contrast was also high for Sb and Sn (3 to 5 times higher) and also significant, though less pronounced (< 2 times higher) for other steel-related metals, such as V, Ni, Mn, or Mo. These differences between metals may reflect variations in background levels and exposure intensities, but they are also attributable to differences in toxicokinetic behavior, because not all metals are excreted equally in urine. In contrast, urine concentrations were similar for metals not associated with steel, such as As or Pb, which are more dependent on diet or other sources of pollution.

The exposure to Cr in many of the working children (geometric mean, 30.1 µg/g creatinine; 25th–75th percentile: 12.3–78.2, or 23.0 µg/L; 25th–75th percentile: 8.38–58.6) was well above the biological exposure limit (BEI) of 25 µg/L for adult workers (at the end of shift at the end of a work week) (American Conference of Governmental Industrial Hygienists 2006). We do not know the species of Cr to which the children were exposed. No BEIs have been established for most of the other metals. However, based on the nature of their work, the absence of occupational hygiene measures, and our observations of the dustiness of the workplaces, most children were probably exposed above occupational limits. This remains to be documented.

Because Cr and Ni are recognized carcinogens (International Agency for Research on Cancer 1990; Klein and Costa 2007; Langård and Costa 2007) and because several metals have pro-oxidant properties, we also measured the urinary concentration of 8-OHdG as an index of oxidative DNA damage (Valavanidis et al. 2009). No significant difference was found between working children and schoolchildren for this parameter. However, we found a highly significant correlation between the concentrations of 8-OHdG and Ni (*r* = 0.41, *p* 0.0001), and also between 8-OHdG and our composite metal exposure index (*r* = 0.46, *p* < 0.001). Others have measured urinary 8-OHdG in children and found that subjects with high urinary As and Cr had the highest level of 8-OHdG (Wong et al. 2005). To the extent that 8-OHdG is predictive of the risk of cancer, our findings indicate that those children with the highest exposure to metals may be at a higher risk of future cancer.

## Conclusions

Our study has shown that the children working in surgical instruments manufacturing units in Sialkot are engaged in hazardous work, as demonstrated by strong evidence of high metal exposure. Although our cross-sectional study did not identify any serious clinical consequences, we cannot rule out the possibility of adverse health effects among children who have discontinued work in this industry. Moreover, we may anticipate that with longer exposure, and even without further exposure, some children will develop (occupational) illnesses in adulthood.

Child labor is a complex societal issue, and the purpose of our research is not to stigmatize those families who send their children to work to improve their living conditions. However, the least that should be done is to prevent the worst excesses and the most hazardous working conditions by implementing—and enforcing—elementary hygiene measures. The companies, health institutions, and countries that import surgical instruments should be aware of the conditions under which children work to make these instruments.

## Supplemental Material

(156 kB) PDF
