# Supplementary material for: Adverse Health Effects of Child Labor: High Exposure to Chromium and Oxidative DNA Damage in Children Manufacturing Surgical Instruments
Source: Environ Health Perspect. 2012 Jun 1;120(10):1469–74. doi: 10.1289/ehp.1104678 (PMC3491927; doi:10.1289/ehp.1104678)
Supplement: (156 kB) PDF [file ehp.1104678.s001.pdf]

Supplemental Material

Adverse Health Effects of Child Labor: High Exposure to Chromium and Oxidative DNA Damage in Children Manufacturing Surgical Instruments

Authors: Muhammad Sughis[1,2,3], Tim S. Nawrot[1,4], Vincent Haufroid[5], Benoit Nemery[1]*

[1] Lung Toxicology Research Unit, Department of Public Health, KU Leuven, Herestraat 49 (706), B-3000 Leuven, Belgium;
[2]Centre of Research for Public Health, Lahore, Pakistan
[3]Lahore College of Pharmaceutical Sciences, Lahore, Pakistan
[4] Centre for Environmental Sciences, Hasselt University, Diepenbeek, Belgium
[5] Louvain Centre for Toxicology and Applied Pharmacology (LTAP) (Université Catholique de Louvain), Brussels, Belgium

Institution where this research was performed:
Lung Toxicology Research Unit, Department of Public Health, KU Leuven, Herestraat 49 (706), B-3000 Leuven, Belgium

*Corresponding author:
Prof. Benoit Nemery
KU Leuven, Department of Public Health,
Laboratorium voor Pneumologie (Longtoxicologie),
Herestraat 49, O&N1:706, B-3000
Leuven, Belgium
E-mail ben.nemery@med.kuleuven.be
Telephone: +32 16330801, Fax: +32 16 330806

Table of Contents Page

Supplemental Material, Table S1
Correlation coefficient of urinary metals in the entire study population 02

Supplemental Material, Table S2
Principal component loadings of the variables 03

Supplemental Material, Table S3
Change in health outcome for a doubling in metal concentrations and for 1 unit change in composite metal exposure index 04

Supplemental Material, Table S1: Correlation coefficient of urinary metals (corrected for creatinine) in the entire study population (n=168)

	**Al**	**V**	**Cr**	**Mn**	**Co**	**Ni**	**Cu**	**Zn**	**As**	**Se**	**Mo**	**Cd**	**Ba**	**Pb**	**U**
**Al**	1														
**V**	0.53	1													
**Cr**	0.42	0.55	1												
**Mn**	0.66	0.71	0.40	1											
**Co**	0.45	0.45	0.34	0.38	1										
**Ni**	0.57	0.72	0.60	0.57	0.74	1									
**Cu**	0.55	0.56	0.42	0.58	0.55	0.69	1								
**Zn**	0.29	0.30	0.08	0.36	0.39	0.41	0.58	1							
**As**	0.37	0.34	0.20	0.30	0.53	0.50	0.58	0.44	1						
**Se**	0.46	0.45	0.38	0.35	0.63	0.67	0.70	0.52	0.66	1					
**Mo**	0.32	0.43	0.42	0.24	0.52	0.66	0.48	0.29	0.45	0.61	1				
**Cd**	0.46	0.48	0.25	0.36	0.59	0.65	0.64	0.45	0.63	0.65	0.78	1			
**Ba**	0.34	0.47	0.02	0.57	0.23	0.29	0.41	0.49	0.22	0.22	0.16	0.28	1		
**Pb**	0.56	0.42	0.16	0.54	0.44	0.49	0.58	0.43	0.51	0.44	0.32	0.57	0.40	1	
**U**	0.28	0.47	0.28	0.58	0.25	0.40	0.51	0.49	0.33	0.35	0.29	0.31	0.55	0.36	1

p-value for the correlations between all metals is significant, except for Cr vs. Zn (p=0.2) and Cr vs. Ba (p=0.7).

Supplemental Material, Table S2: Principal component loadings of the variables

Metal	Correlations
Aluminum	0.68
Vanadium	0.81
Chrome	0.72
Manganese	0.74
Nickel	0.88
Copper	0.79
Molybdenum	0.69
Cadmium	0.72
Tin	0.63
Antimony	0.78
Uranium	0.56

The composite exposure index (principal component 1) obtained by the first component of a principal components analysis of the urinary concentration of 11 metals (Al, V, Cr, Mn, Ni, Cu, Mo, Cd, Sn, Sb, and U) explained 54% of total variance (eigenvalue 5.97). The results of principal component were significantly higher in the group of working children (explained 27% of the variance).

Supplemental Material, Table S3: Change in health outcome for a doubling in metal concentrations and for 1 unit change in composite metal exposure index

**Characteristics**	**Cr**	**Ni**	**As**	**Cd**	**Exposure index**[a]
Systolic BP – mmHg	0.27 (-0.10 to 0.65)	0.57 (-0.75 to 1.90)	1.43 (-0.30 to 3.17)	0.91 (-0.58 to 2.41)	0.37 (-0.14 to 0.89)
Diastolic BP – mmHg	0.07 (-0.33 to 0.47)	-0.08 (-1.50 to 1.33)	0.63 (-1.23 to 2.50)	0.08 (-1.51 to 1.70)	0.03 (-0.51 to 0.59)
FVC – L	0.07 (0.04 to 0.10)**	0.03 (-0.11 to 0.18)	-0.09 (-0.29 to 0.09)	-0.02 (-0.18 to 0.13)	0.02 (-0.01 to 0.06)
FEV1 – L	0.06 (0.04 to 0.09)**	-0.004 (-0.12 to 0.11)	-0.15 (-0.31 to 0.005)	-0.05 (-0.18 to 0.07)	0.01 (-0.01 to 0.04)
FEV1/FVCx100	0.001 (-0.44 to 0.44)	-1.32 (-3.20 to 0.54)	-2.52 (-4.93 to -0.11)*	-1.45 (-3.46 to 0.55)	-0.15 (-0.35 to 0.04)
Physician Reported Asthma	1.22 (0.98 to 1.51)	0.67 (0.34 to 1.30)	0.75 (0.34 to 1.85)	0.56 (0.24 to 1.30)	1.04 (0.80 to 1.34)
8-OHdG	1.03 (0.99 to 1.07)	1.40 (1.23 to 1.61)**	1.13 (0.92 to 1.40)	1.22 (1.05 to 1.43)*	0.05 (0.03 to 0.07)**

§The data is mentioned as regression coefficients or odds ratios (asthma) with 95% CI for a doubling in the urinary metal concentration (µg/g creatinine). Regressions with systolic and diastolic BP adjusted for age; with FVC and FEV1 adjusted for height; 8-OHdG adjusted for age, height, and weight.

[a] Changes for 1 unit increase in exposure score (principal component), obtained as described in Supplemental Table S2.

*p<0.05
**p<0.0001
